# Quantifying the intensity of permethrin insecticide resistance in *Anopheles* mosquitoes in western Kenya

**DOI:** 10.1186/s13071-017-2489-6

**Published:** 2017-11-06

**Authors:** Seline Omondi, Wolfgang Richard Mukabana, Eric Ochomo, Margaret Muchoki, Brigid Kemei, Charles Mbogo, Nabie Bayoh

**Affiliations:** 1 School of Biological Sciences, University of Nairobi, P.O Box 30197-00100, Nairobi, Kenya; 2 Kenya Medical Research Institute (KEMRI), P.O Box 1578-40100, Kisumu, Kenya; 3 Science for Health, P.O Box 44970-00100, Nairobi, Kenya; 4 KEMRI-Centre for Geographic Medicine Research-Coast, P.O Box 230-80108, Kilifi, Kenya; 5 KEMRI-Wellcome Trust Research Program, P.O Box 43640-00100, Nairobi, Kenya; 6 US Centers for Disease Control and Prevention-Kenya, P.O Box 1578-40100, Kisumu, Kenya

**Keywords:** Insecticide resistance, Intensity, Permethrin, *Anopheles gambiae*

## Abstract

**Background:**

The development and spread of resistance among local vectors to the major classes of insecticides used in Long-Lasting Insecticidal Nets (LLINs) and Indoor Residual Spraying (IRS) poses a major challenge to malaria vector control programs worldwide. The main methods of evaluating insecticide resistance in malaria vectors are the WHO tube bioassay and CDC bottle assays, with their weakness being determination of resistance at a fixed dose for variable populations. The CDC bottle assay using different insecticide dosages has proved applicable in ascertaining the intensity of resistance.

**Methods:**

We determined the status and intensity of permethrin resistance and investigated the efficacy of commonly used LLINs (PermaNet® 2.0, PermaNet® 3.0 and Olyset®) against 3–5 day-old adult female *Anopheles* mosquitoes from four sub-counties; Teso, Bondo, Rachuonyo and Nyando in western Kenya. Knockdown was assessed to 4 doses of permethrin; 1× (21.5 μg/ml), 2× (43 μg/ml), 5× (107.5 μg/ml) and 10× (215 μg/ml) using CDC bottle assays.

**Results:**

Mortality for 0.75% permethrin ranged from 23.5% to 96.1% in the WHO tube assay. Intensity of permethrin resistance was highest in Barkanyango Bondo, with 84% knockdown at the 30 min diagnostic time when exposed to the 10× dose. When exposed to the LLINs, mortality ranged between— 0–39% for Olyset®, 12–88% for PermaNet® 2.0 and 26–89% for PermaNet® 3.0. The efficacy of nets was reduced in Bondo and Teso. Results from this study show that there was confirmed resistance in all the sites; however, intensity assays were able to differentiate Bondo and Teso as the sites with the highest levels of resistance, which coincidentally were the two sub-counties with reduced net efficacy.

**Conclusions:**

There was a reduced efficacy of nets in areas with high resistance portraying that at certain intensities of resistance, vector control using LLINs may be compromised. It is necessary to incorporate intensity assays in order to determine the extent of threat that resistance poses to malaria control.

**Electronic supplementary material:**

The online version of this article (10.1186/s13071-017-2489-6) contains supplementary material, which is available to authorized users.

## Background

Malaria is the most detrimental parasitic infection worldwide accounting for 429,000 deaths and 212 million cases in 2015 [1]. Globally, malaria cases have decreased since the year 2000 but it is still considered a life threatening disease which takes a life of at least one child every two minutes [2]. The greatest contributor to this decline was the use of long lasting insecticidal nets (LLINs), effective anti-malarial treatments and indoor residual spraying (IRS) [3]. Despite the national and global efforts, the disease still remains a burden, which can partly be attributed to the possible failure of vector control interventions as a result of the development of insecticide resistance among vectors [4–6]. Sustained exposure of mosquitoes to these insecticides, and agricultural activities and industrial pollutants [7], have led to the development of insecticide resistance which in turn threatens the robustness of the vector control interventions in place [8].

Insecticide resistance has been reported in almost all the countries endemic for malaria in sub-Saharan Africa [9] (http://www.irmapper.com//) with resistance to the pyrethroids, which is the only class of insecticides recommended by the WHO on Insecticidal Treated Nets (ITNs) [2]. The use of ITNs has contributed immensely to the success of malaria reduction and these drawbacks will hinder its functionality in curbing malaria transmission by *Anopheles* mosquitoes. The only action is therefore to adapt a proactive approach and modify current practices so as to delay the spread of resistance and preserve the effectiveness of current insecticides at least until new ones are developed. To do this, regions endemic for malaria should regularly monitor the existence and intensity of insecticide resistance that may affect the efficacy of the insecticide based vector control interventions in reducing the burden of malaria.

Phenotypic resistance can be evaluated using the WHO susceptibility test and the CDC bottle assays [10, 11]. Until the time this study was conducted, the WHO susceptibility test only provided information on whether a particular mosquito population is susceptible or resistant at the 90% level of mortality and did not provide much detail on varying levels of intensity of resistance. For example, a population with 10% mortality to an insecticide and another with 89% mortality to the same insecticide were both classified as resistant, despite the fact that, programmatically, the two levels were bound to have different levels of impact to malaria control. For instance western Kenya has been reported to have high frequencies of pyrethroid resistance [12] but the intensities of this resistance, and whether or not it impacts on the burden, is not known. The majority of ITNs in western Kenya are impregnated with permethrin or deltamethrin. Analysis performed on mortality data for deltamethrin and permethrin have indicated the two are positively correlated [12] and only one insecticide was used for this study. This study aimed to evaluate the intensity of permethrin resistance in four regions and its impact on LLINs, a major vector control intervention.

## Methods

### Study site

The study was conducted in four malaria endemic sub-counties in western Kenya: Bondo (0°14′N, 34°16′E), Rachuonyo (0°30′S, 34°43′E), Nyando (0°11′S, 34°55′E) and Teso (0°43′N, 34°43′E). Malaria is endemic with high and perennial seasonal peaks between April to July and November to December [13]. Nyando and Rachuonyo have benefitted from both IRS and ITNs whereas in Teso and Bondo only ITNs have been deployed. These sites have been described previously [12].

Mass distribution of LLINs was conducted in the year 2006, with either Olyset (Sumitomo chemicals, Tokyo, Japan) or Permanet 2.0 (Vestergaard Frandsen, Lausanne, Switzerland) nets in Kenya [14]. In 2008 and 2009 IRS first and second rounds were conducted in Rachuonyo using Lamda cyhalothrin (ICON CS, Syngenta, Basel, Switzerland) and Alphacyhalothrin (Fendona, BASF, Auckland, New Zealand) respectively [14]. In 2010 both Nyando and Rachuonyo were targeted for IRS and this was conducted using deltamethrin (K-Othrine, Bayer, Selangor Darul Ehsan, Malaysia), a repeat was conducted in 2011 with lambda cyhalothrin (ICON CS, Syngenta, Basel, Switzerland) in the two sub-counties [15].

### Mosquito collection

*Anopheles gambiae* (*s.l.*) larvae were sampled from October 2015 to February 2016 from two sub-locations per sub-county: Akiriamasi and Kaliwa in Teso sub-county; Kamenya Central and Kobuya in Rachuonyo sub-county; Kochogo North and Ahero in Nyando sub-county; and Barkanyango and Omia Mwalo in Bondo sub-county. Mosquitoes were reared in the KEMRI CGHR insectary to adult stage using methods described previously [12] and female mosquitoes of age between 3 and 5 days were subjected to different bioassays.

### WHO tube assays

WHO susceptibility test were performed according to WHO guidelines [10] using permethrin (0.75%) impregnated papers obtained from Universiti Sains Malaysia. Exposure time was 1 h, where knockdown was recorded at an interval of 10 min after which the mosquitoes were transferred to the holding tube, provided with 10% sugar solution and mortality recorded after 24 h.

### CDC bottle assay

Four different vials containing permethrin insecticide with different diagnostic doses (1×, 2×, 5× and 10×) were provided by the CDC, Atlanta. These were diluted in acetone (48 ml) to provide a stock solution with a concentration of 21.5 μg/ml, 43 μg/ml, 107.5 μg/ml and 215 μg/ml, respectively, for each diagnostic dose. A solution of 1 ml was added to a 250 ml Wheaton bottle left to dry overnight after which mosquitoes were exposed for 2 h, then the experiment was discontinued. Knockdown was recorded at an interval of 10 min.

### Cone bioassay

Cone bioassays were performed using 25 × 25 cm (L × W) pieces of new PermaNet® 2.0, PermaNet® 3.0 and Olyset® nets provided by the manufacturers. PermaNet® 2.0 and PermaNet® 3.0 were provided by Vestergaard Frandsen while Olyset® net were provided by Sumitomo chemicals. According to the manufacturer, the fabrics were impregnated with 55 mg/m^2^ deltamethrin for PermaNet® 2.0 and 20 g/kg permethrin for Olyset® net. PermaNet® 3.0 was impregnated with deltamethrin and an additional synergist, piperonylbutoxide (PBO) was incorporated on the upper square. The roof contained 25 g/kg ± 25% of PBO in addition to 4 g/kg ± 25% of deltamethrin while the side panels were impregnated with 2.8 g/kg ± 25% deltamethrin. Five net pieces were cut from each net type (four from the side panels and one from the roof) and used in the assays. Approximately 20 mosquitoes were exposed to each net piece. Exposure time was 3 min with the cone slanted at an angle of 45°; mosquitoes were then transferred to paper cups and knockdown monitored at an interval of 10 min for 1 h. Mortality was recorded after 24 h.

### Data analysis

Mortality was scored according to the WHO criteria with a percentage mortality of 98–100% termed as susceptible, between 90 and 97% indicating the possibility of resistance and less than 90% indicating resistance [10]. In order to determine the significant difference in the susceptibility of *Anopheles* mosquitoes, percentage mean mortalities were compared between the four sub-counties using one way ANOVA in SPSS v20. For the intensity (CDC bottle) assays probit analysis was used to calculate knockdown rates at KDT50 for the different concentrations of permethrin at 95% confidence interval (SPSS v20). The efficacy of nets was determined using the WHO criteria where by the knockdown and mortality, 95 and 80%, respectively, determined the threshold in the exposed mosquitoes. Mortality rates for the three net types were compared using ANOVA with Tukey’s HSD *post-hoc* test, while top and side panel for PermaNet® was done using Student’s t-test (SPSS v20). Statistical significance was assessed at an alpha value of 0.05.

## Results

A total of 5291 female *Anopheles* mosquitoes were exposed to the three different assays. For the WHO tube assays, 826 female mosquitoes from eight sites were exposed to permethrin coated papers. For the CDC bottle assays and cone assays, a total of 2319 and 2146 female mosquitoes were exposed, respectively.

### WHO susceptibly assays

Permethrin resistance was observed in the majority of the sites where the collections were done. There were no statistical significant differences in mean mortalities between the sub-counties (*F*_(3,4)_ = 2.668, *P* = 0.183). Mortality rates varied from site to site, with the least susceptibility of 23% in Kaliwa, Teso sub-county, and a possible resistance of 96% in Kochogo, Nyando sub-county (Table 1).

**Table 1:** Susceptibility of *An. gambiae* (*s.l.*) to permethrin 0.75%, with 24-h mortality, KDT50 and KDT99 at 95% confidence interval in all the study sites

Sub-county	Site	Total tested	24-h mortality (%)	KDT50 (95% CI) min	KDT99 (95% CI) min
Bondo	Barkanyango	121	28.9	252.69 (141.84–1004.13)	6725.14 (1451.65–283,549.49)
	Omia Mwalo	96	74	46.78 (40.14–58.07)	156.94 (102.64–439.03)
Teso	Kaliwa	81	23.5	164.29 (94.28–7725.93)	18,767.37 (2412.58–596,742.22)
	Akiriamas	100	53	54.08 (47.01–65.51)	698.27 (391.13–1764.71)
Rachuonyo	Kobuya	100	63	33.82 (24.06–45.42)	106.12 (66.88–595.50)
	Kamenya	99	76.8	47.92 (41.91–57.92)	127.57 (89.47–312.97)
Nyando	Ahero	126	86.5	46.39 (37.83–64.14)	152.75 (92.52–841.02)
	Kochogo	102	96.1	41.46 (31.80–57.53)	115.78 (73.36–869.11)
Kisumu strain		74	98.6	29.64 (23.57–35.82)	101.66 (70.47–232.87)

*Abbreviations*: *KDT50* time required for 50% of mosquitoes exposed to the insecticide to be knocked-down, *KDT99* time required for 99% of mosquitoes exposed to the insecticide to be knocked-down

### CDC bottle assay

**Fig. 1:**
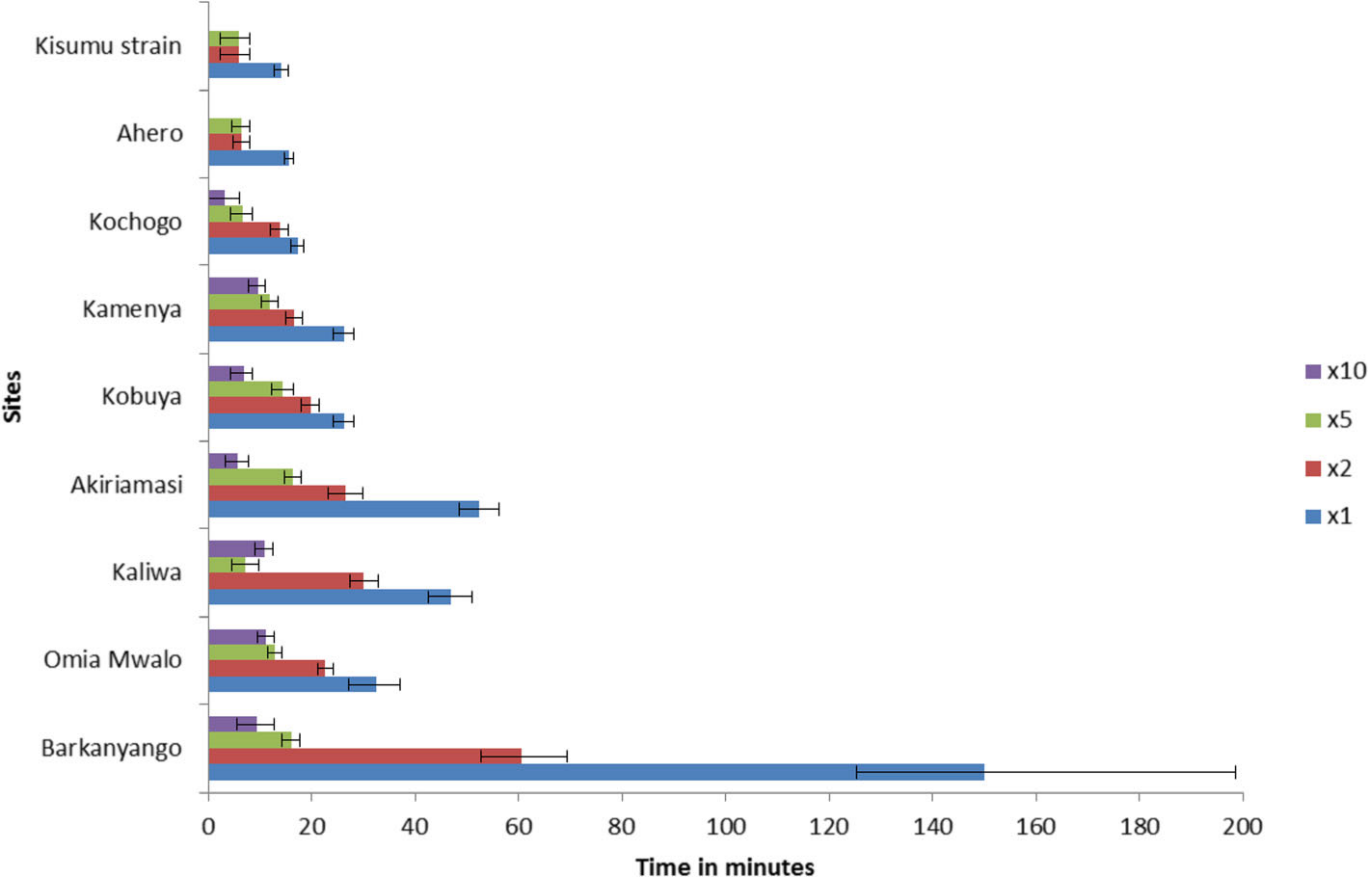
KDT50 values at 95% CI for female *Anopheles* mosquitoes exposed to 1×, 2×, 5× and 10× permethrin concentrations in the CDC bottle bioassay. KDT50 represents the time required for 50% of mosquitoes exposed to the insecticide to be knocked-down

Teso and Bondo showed lower knockdown rates to all the four doses of permethrin. Keeping a fixed time of 2 h but using different concentrations, the KDT50 at 95% confidence interval was lowest in Nyando and highest in Barkanyango, Bondo with the rest of the sites having overlapping values (Fig. 1).

### Cone bioassay

**Fig. 2:**
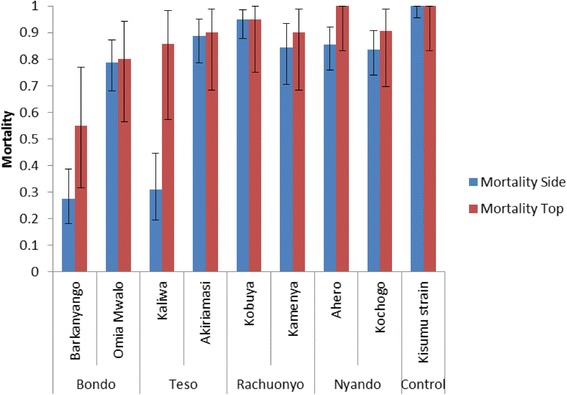
Proportion mortality for PermaNet® 3.0 top and side panels against field collected *An*. *gambiae* (*s.l.*)

Comparison among the three LLINs had a significant influence on mortality rates *F*_(2,21)_ = 16.726, *P* < 0.001. However, Tukey’s HSD *post-hoc* test showed there was no significant difference between PermaNet® 2.0 and PermaNet® 3.0 (*M* = 8.04, *P* = 0.697), but one was observed between Olyset® net and the two PermaNet nets with significant values (*M* = 53.01, *P* < 0.001 and *M* = 44.97, *P* < 0.001 for PermaNet® 2.0 and PermaNet® 3.0, respectively). Mortalities ranged between 26 and 89% for PermaNet® 3.0 (net pieces from the sides and top panel) and between 12 and 88% for PermaNet® 2.0 while Olyset® recorded the lowest mortalities of 0–39% (Table 2). Mortalities observed against the side and top panel for PermaNet® 3.0 ranged from 27.5–88.6% and 55–100%, respectively (*t*_(7)_ = -2.106, *P* = 0.073) (Fig. 2).

**Table 2:** Knockdown (KD) and 24-h mortality rates of *Anopheles gambiae* (*s.l.*) mosquitoes exposed to PermaNet® 2.0, PermaNet® 3.0 and Olyset®

Sub-county	Site	Net type								
		PermaNet® 2.0			PermaNet® 3.0			Olyset®		
		*n*	KD (%)	Dead (%)	*n*	KD (%)	Dead (%)	*n*	KD (%)	Dead (%)
Bondo	Barkanyango	95	61	12	100	90	26	58	17	9
	Omia Mwalo	100	94	51	100	97	73	99	35	23
Teso	Kaliwa	75	92	47	72	97	42	60	23	0
	Akiriamasi	140	89	70	90	99	89	65	20	17
Rachuonyo	Kobuya	103	97	69	100	100	77	103	63	39
	Kamenya	77	95	77	65	97	86	62	52	23
Nyando	Ahero	100	99	75	102	100	69	111	47	11
	Kochogo	100	99	88	106	100	68	103	41	27
Kisumu Strain		100	100	100	100	100	100	100	96	84

*Abbreviations*: *n* number tested, *KD* knocked-down mosquitoes at 60 min, *Dead* mortality after 24 h

### Molecular assays

**Fig. 3:**
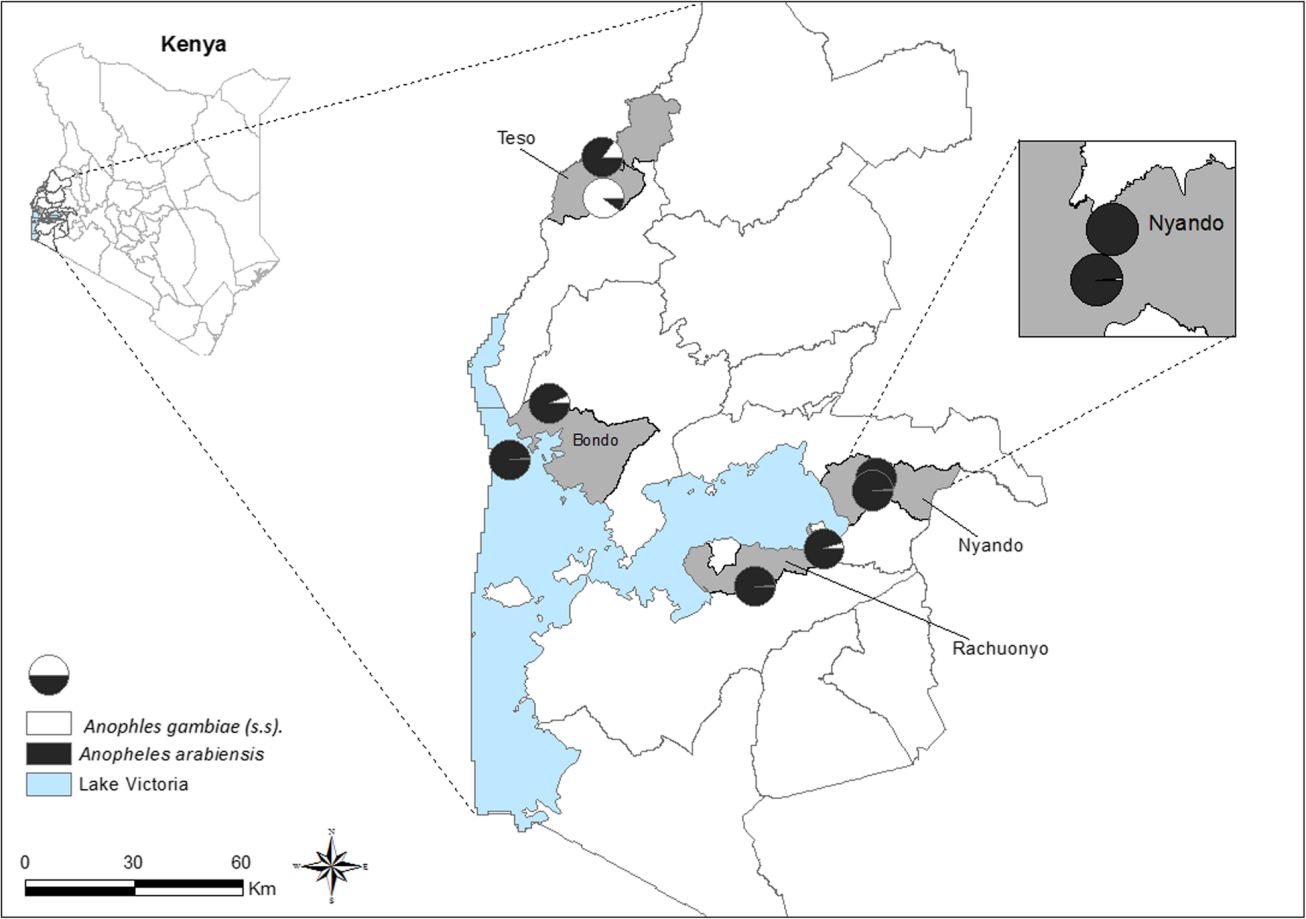
Map of Kenya showing the distribution of *Anopheles gambiae* (*s.s.*) and *An. arabiensis* in the four sub-counties

*Anopheles arabiensis* was the major species at the majority of the sites, while it was only in Kaliwa, Teso that *An. gambiae* (*s.s.*) was the dominant species (Fig. 3).

## Discussion

The overall objective of the study was to quantify the intensity of permethrin resistance in western Kenya; it revealed that despite the presence of permethrin resistance in all the sites, the frequency of resistance varied from site to site. At the sites that depicted higher intensities of resistance to the insecticide, the mosquitoes also recorded lower levels of mortalities when exposed to the commonly used LLINs.

Resistance to malaria vectors to the major classes of insecticides is a potential threat that, with time, may contribute to absolute failure of the control interventions that are already in place. A proactive approach should be adopted so as to delay the spread or arrest resistance in areas with mutations deterring the effectiveness of the already available insecticides. Based on the WHO susceptibility assay and CDC bottles bioassay, insecticide resistance in malaria vectors have been reported in 64 malaria endemic countries [9] (http://www.irmapper.com//), the African continent having majority of the cases. Susceptibility assays done using the WHO test kits is essential for testing the status of resistance while CDC bottle bioassays with added modifications; using different diagnostic doses provides information on the level of resistance. Mosquitoes collected from two different sites might yield a mortality of 15% and 86%, the WHO susceptibility test, as was stipulated at the time this study was conducted, would deem both populations as resistance. By use of escalated insecticide concentration in the bottle bioassays the two populations are treated differently and this gives more knowledge on the extremes of the resistance. Barkanyango was ranked with the highest level of insecticide using the 1×, but the results overlap with those of the other sites when different dosages of permethrin are used. The high intensities of resistance in Bondo may be due to the use of LLINs, which started in the early 2000s [16]. Another site with relatively high proportions of insecticide resistance was Teso, this sub-county borders Tororo in Uganda, where cotton farming is the major economic activity. Evidence shows that mosquitoes collected from cotton cultivation sites have increased insecticide resistance [7]. The use of insecticides both across the border and for tobacco in Teso might offer selection pressure for the vectors and, in addition to this, the proximity of the sub-county to Uganda, where the first incidence of both *kdr* genes were identified, may have contributed to a gene flow [17]. Unlike Bondo, Rachuonyo and Nyando, which had minimal levels of resistance, LLINs coverage in the sub-counties was introduced in the late 2000s.

The *An. gambie* (*s.l*.) population from the four sub-counties in western Kenya was classified as resistant to permethrin, with the exception of Kochogo in Nyando. Resistance to the pyrethroids has been linked to the *kdr* genetic mutation and in 2015 both *kdr* east (L104S) and *kdr* west (L104F) were observed in western Kenya [18]. The occurrence of both genes in this region might have impacted on the pyrethroids resistance level. The majority of the malaria vectors in this area belong to the *An. gambiae* complex and the *An. funestus* group [19]. The population used in the assays were mainly composed of *An. gambiae* sub-species; *An. arabiensis* and to a lesser extent *An. gambiae* (*s.s.*). Only Kaliwa, Teso had *An. gambiae* (*s.s.*) as the main species and the area recorded the lowest mortality (23%), an occurrence that may be attributed to the presence of *An. gambiae* (*s.s.*) as the main species [20]. The decline in the *An*. *gambiae* (*s.s.*) species in Teso may be attributed to the intense use of LLINs since these species are majorly anthropophagic [16], while an increased in *An. arabiensis*, the dominant species in Nyando, Rachuonyo and Bondo, is due to the fact that this species are more zoophilic and exophilic in nature thereby evading the indoor control interventions (both IRS and LLINs).

When cone assays were conducted Barkanyango, Bondo had both lower knockdown and mortality rates, which indicates there is likely to be a control failure compared to the rest of the sites given the varying levels of intensities observed. The results indicated that the performance of Olyset® nets compared to PermaNet® did poorly in the cone assays with the field collected mosquitoes. These findings are similar to those of Benin, where high resistance to permethrin attributed to lowered mortalities of the same populations to permethrin treated Olyset® net [21, 22]. PermaNet® 3.0 produced higher mortalities in majority of the sites; the net contains deltamethrin and piperonyl butoxide (PBO). We were unable to obtain Olyset® plus nets, impregnated with permethrin and PBO, for this study. The synergist, (PBO), works by enhancing the effect of pyrethroids via inhibition of metabolic enzymes in the mosquitoes, rendering them susceptible to the pyrethroid and the organochloride insecticides [23]. Previous studies, which led to the production of synergist LLINs, deduced that mosquitoes make contact with the top panel first, thereby making it appropriate to only incorporate this chemical on the upper part of the nets [24]. The biology behind it is that the nets acts as a chimney and host volatiles and heat produced are funneled upwards [24]. PermaNet® 2.0 on the other hand lacks PBO, but is preferred in sub-Saharan Africa because they are cheaper compared to PermaNet®3.0.

Finally, there is need for a crucial resistance management strategy, due to great concerns brought about by failure of the control interventions in place. Bioassays are only a forefront tool for detection of resistance, after which, a strategy or a control tool can be implemented. The choice of the assay is very important and relies on the objectives and results to be obtained. The CDC bioassay only scores knockdown but the flexibility of using different insecticide concentrations is an added advantage (a crucial factor in measuring resistance intensity). Cone assays on the other hand are recommended in assessing the response of mosquitoes to the field dose and formulations of insecticides.

## Conclusions

The study confirmed resistance in all the sites, with an exception of one possibility of resistance; however, intensity assays were able to differentiate the sites with high resistance. These sites coincidentally had reduced net efficacy, deducing that at different intensities of resistance, vector control using LLINs may be compromised. Therefore, it is necessary to incorporate intensity assays in order to determine the extent of the threat that resistance poses to malaria control.

## Additional file

Additional file 1: Table S1. Detailed results of the 2 h CDC bottle bioassays performed for the different permethrin concentration in the four sub-counties in western Kenya. (DOCX 16 kb)

## Abbreviations

CDC: Centers for Disease Control and Prevention
CGHR: Centre for Global Health Research
IRS: Indoor Residual Spraying
ITNs: Insecticide Treated Nets
*Kdr*: Knockdown resistance
KEMRI: Kenya Medical Research Institute
LLINs: Long-lasting insecticidal nets
PBO: Piperonyl butoxide
WHO: World Health Organization
