# Supplementary material for: Quantifying the intensity of permethrin insecticide resistance in Anopheles mosquitoes in western Kenya
Source: Parasit Vectors. 2017 Nov 6;10:548. doi: 10.1186/s13071-017-2489-6 (PMC5674850; doi:10.1186/s13071-017-2489-6)
Supplement: Additional file 1: Table S1. — Detailed results of the 2 h CDC bottle bioassays performed for the different permethrin concentration in the four sub-counties in western Kenya. (DOCX 16 kb) [file 13071_2017_2489_MOESM1_ESM.docx]

| **Additional file 1: Table S1.** Detailed results of the 2 h CDC bottle bioassays performed for the different permethrin concentration in the four sub-counties in western Kenya | | | | | | | | | | | | | | | | |
| --- | --- | --- | --- | --- | --- | --- | --- | --- | --- | --- | --- | --- | --- | --- | --- | --- |
|  |  |  |  | Knock Down time (0-120)minutes | | | | | | | | | | | | |
| Sub County | Site | Permethrin Dosage | Number tested | 0 | 10 | 20 | 30 | 40 | 50 | 60 | 70 | 80 | 90 | 100 | 110 | 120 |
| Bondo | Barkanyango | ×1 | 55 | 0 | 0 | 1 | 4 | 4 | 5 | 11 | 14 | 16 | 18 | 19 | 20 | 20 |
| Bondo | Barkanyango | ×2 | 103 | 0 | 6 | 14 | 17 | 21 | 41 | 47 | 51 | 59 | 73 | 78 | 85 | 90 |
| Bondo | Barkanyango | ×5 | 84 | 0 | 22 | 53 | 68 | 75 | 79 | 82 | 84 | 84 | 84 | 84 | 84 | 84 |
| Bondo | Barkanyango | ×10 | 64 | 0 | 38 | 51 | 54 | 55 | 64 | 64 | 64 | 64 | 64 | 64 | 64 | 64 |
| Bondo | Omia Mwalo | ×1 | 62 | 0 | 1 | 6 | 30 | 44 | 48 | 59 | 59 | 59 | 59 | 59 | 59 | 59 |
| Bondo | Omia Mwalo | ×2 | 98 | 0 | 3 | 39 | 72 | 85 | 96 | 98 | 98 | 98 | 98 | 98 | 98 | 98 |
| Bondo | Omia Mwalo | ×5 | 61 | 0 | 18 | 50 | 60 | 61 | 61 | 61 | 61 | 61 | 61 | 61 | 61 | 61 |
| Bondo | Omia Mwalo | ×10 | 60 | 0 | 21 | 57 | 59 | 60 | 60 | 60 | 60 | 60 | 60 | 60 | 60 | 60 |
| Teso | Akiriamasi | ×1 | 60 | 0 | 1 | 6 | 14 | 19 | 23 | 29 | 40 | 46 | 48 | 50 | 54 | 57 |
| Teso | Akiriamasi | ×2 | 66 | 0 | 7 | 19 | 30 | 48 | 57 | 62 | 64 | 65 | 66 | 66 | 66 | 66 |
| Teso | Akiriamasi | ×5 | 65 | 0 | 11 | 39 | 59 | 64 | 65 | 65 | 65 | 65 | 65 | 65 | 65 | 65 |
| Teso | Akiriamasi | ×10 | 63 | 0 | 46 | 60 | 62 | 63 | 63 | 63 | 63 | 63 | 63 | 63 | 63 | 63 |
| Teso | Kaliwa | ×1 | 59 | 0 | 1 | 9 | 21 | 25 | 29 | 36 | 42 | 44 | 45 | 47 | 50 | 53 |
| Teso | Kaliwa | ×2 | 46 | 0 | 0 | 12 | 26 | 29 | 35 | 42 | 45 | 46 | 46 | 46 | 46 | 46 |
| Teso | Kaliwa | ×5 | 46 | 0 | 30 | 40 | 43 | 44 | 44 | 46 | 46 | 46 | 46 | 46 | 46 | 46 |
| Teso | Kaliwa | ×10 | 60 | 0 | 28 | 49 | 59 | 60 | 60 | 60 | 60 | 60 | 60 | 60 | 60 | 60 |
| Rachuonyo | Kobuya | ×1 | 59 | 0 | 1 | 8 | 43 | 54 | 55 | 57 | 59 | 59 | 59 | 59 | 59 | 59 |
| Rachuonyo | Kobuya | ×2 | 62 | 0 | 5 | 30 | 51 | 57 | 62 | 62 | 62 | 62 | 62 | 62 | 62 | 62 |
| Rachuonyo | Kobuya | ×5 | 45 | 0 | 12 | 30 | 43 | 44 | 44 | 45 | 45 | 45 | 45 | 45 | 45 | 45 |
| Rachuonyo | Kobuya | ×10 | 60 | 0 | 45 | 57 | 60 | 60 | 60 | 60 | 60 | 60 | 60 | 60 | 60 | 60 |
| Rachuonyo | Kamenya | ×1 | 64 | 0 | 2 | 17 | 31 | 57 | 60 | 64 | 64 | 64 | 64 | 64 | 64 | 64 |
| Rachuonyo | Kamenya | ×2 | 62 | 0 | 11 | 36 | 55 | 62 | 62 | 62 | 62 | 62 | 62 | 62 | 62 | 62 |
| Rachuonyo | Kamenya | ×5 | 63 | 0 | 18 | 59 | 63 | 63 | 63 | 63 | 63 | 63 | 63 | 63 | 63 | 63 |
| Rachuonyo | Kamenya | ×10 | 60 | 0 | 29 | 60 | 60 | 60 | 60 | 60 | 60 | 60 | 60 | 60 | 60 | 60 |
| Nyando | Kochogo | ×1 | 115 | 0 | 11 | 71 | 108 | 112 | 113 | 114 | 115 | 115 | 115 | 115 | 115 | 115 |
| Nyando | Kochogo | ×2 | 75 | 0 | 19 | 59 | 68 | 72 | 73 | 73 | 74 | 75 | 75 | 75 | 75 | 75 |
| Nyando | Kochogo | ×5 | 73 | 0 | 49 | 68 | 73 | 73 | 73 | 73 | 73 | 73 | 73 | 73 | 73 | 73 |
| Nyando | Kochogo | ×10 | 57 | 0 | 53 | 56 | 57 | 57 | 57 | 57 | 57 | 57 | 57 | 57 | 57 | 57 |
| Nyando | Ahero | ×1 | 202 | 0 | 40 | 132 | 183 | 199 | 201 | 202 | 202 | 202 | 202 | 202 | 202 | 202 |
| Nyando | Ahero | ×2 | 125 | 0 | 82 | 119 | 125 | 125 | 125 | 125 | 125 | 125 | 125 | 125 | 125 | 125 |
| Nyando | Ahero | ×5 | 91 | 0 | 61 | 88 | 91 | 91 | 91 | 91 | 91 | 91 | 91 | 91 | 91 | 91 |
| Nyando | Ahero | ×10 | 61 | 0 | 61 | 61 | 61 | 61 | 61 | 61 | 61 | 61 | 61 | 61 | 61 | 61 |
| Kisumu strain | | ×1 | 56 | 0 | 11 | 44 | 55 | 56 | 56 | 56 | 56 | 56 | 56 | 56 | 56 | 56 |
| Kisumu strain | | ×2 | 60 | 0 | 50 | 59 | 60 | 60 | 60 | 60 | 60 | 60 | 60 | 60 | 60 | 60 |
| Kisumu strain | | ×5 | 60 | 0 | 56 | 60 | 60 | 60 | 60 | 60 | 60 | 60 | 60 | 60 | 60 | 60 |
| Kisumu strain | | ×10 | 60 | 0 | 60 | 60 | 60 | 60 | 60 | 60 | 60 | 60 | 60 | 60 | 60 | 60 |
